# Intramuscular Soft Tissue Giant Cell Tumor of the Forearm: A Case Report

**DOI:** 10.1002/ccr3.70371

**Published:** 2025-03-28

**Authors:** Kaissar Yammine, Emanuel‐Youssef Dib, Moueen Bou Ghanem

**Affiliations:** ^1^ Department of Orthopedic Surgery, Lebanese American University Medical Center‐Rizk Hospital Lebanese American University School of Medicine Beirut Lebanon; ^2^ Medical Student University of Balamand School of Medicine Balamand Lebanon; ^3^ Department of Pathology, Lebanese American University Medical Center‐Rizk Hospital Lebanese American University School of Medicine Beirut Lebanon

**Keywords:** benign soft tissue tumor, forearm, giant cell tumor of soft tissue, pathology

## Abstract

We report the case of a 33‐year‐old female who presented with a painful, rapidly growing mass on her left dorsal forearm. Physical examination revealed a 2 cm firm, well‐demarcated, non‐erythematous nodule beneath the skin, with an initial diagnosis of lipoma. Upon removal, the mass was found to be intramuscular beneath the antebrachial fascia. The histopathology confirmed a benign giant cell tumor of soft tissue (GCT‐ST). The patient was informed of the potential risk of recurrence, and regular follow‐up was recommended. At the last follow‐up, no clinical evidence of recurrence was noted. This report highlights a rare case of an intramuscular soft tissue GCT at an exceptional location, the forearm.

**Summary**

- This case report described an unusual example of a soft tissue giant cell tumor located at an exceptional location, at the forearm *and* within the muscle.
- The initial diagnosis was lipoma but the pathology revealed a giant cell tumor.
- The follow‐up was uneventful following ‘en bloc’ resection.

## 1 Introduction

Giant cell tumors of soft tissue (GCT‐ST) are extremely rare. They are usually benign but locally aggressive tumors that share histological similarities with giant cell tumors of the bone (GCTB) but are genetically different [1, 2]. It was first described by Salm and Sissons in 1972 [3]. These tumors tend to recur locally (6.2%–21%), but distant metastasis has occurred only in rare cases. GCT‐ST can also be malignant, and some recent cases have been described in the literature [2]. A study on primary giant cell tumors of soft tissue found that these tumors most commonly occur in the lower limbs, followed by the trunk, with the upper limbs being the least frequent location. The tumors generally range in size between 1 and 10 cm and are predominantly superficial [4]. The present case is unique in relation to its anatomical location and site; an intramuscular giant cell tumor of soft tissue located on the dorsal forearm.

## 2 Case Description

### 2.1 initial presentation

**FIGURE 1:**
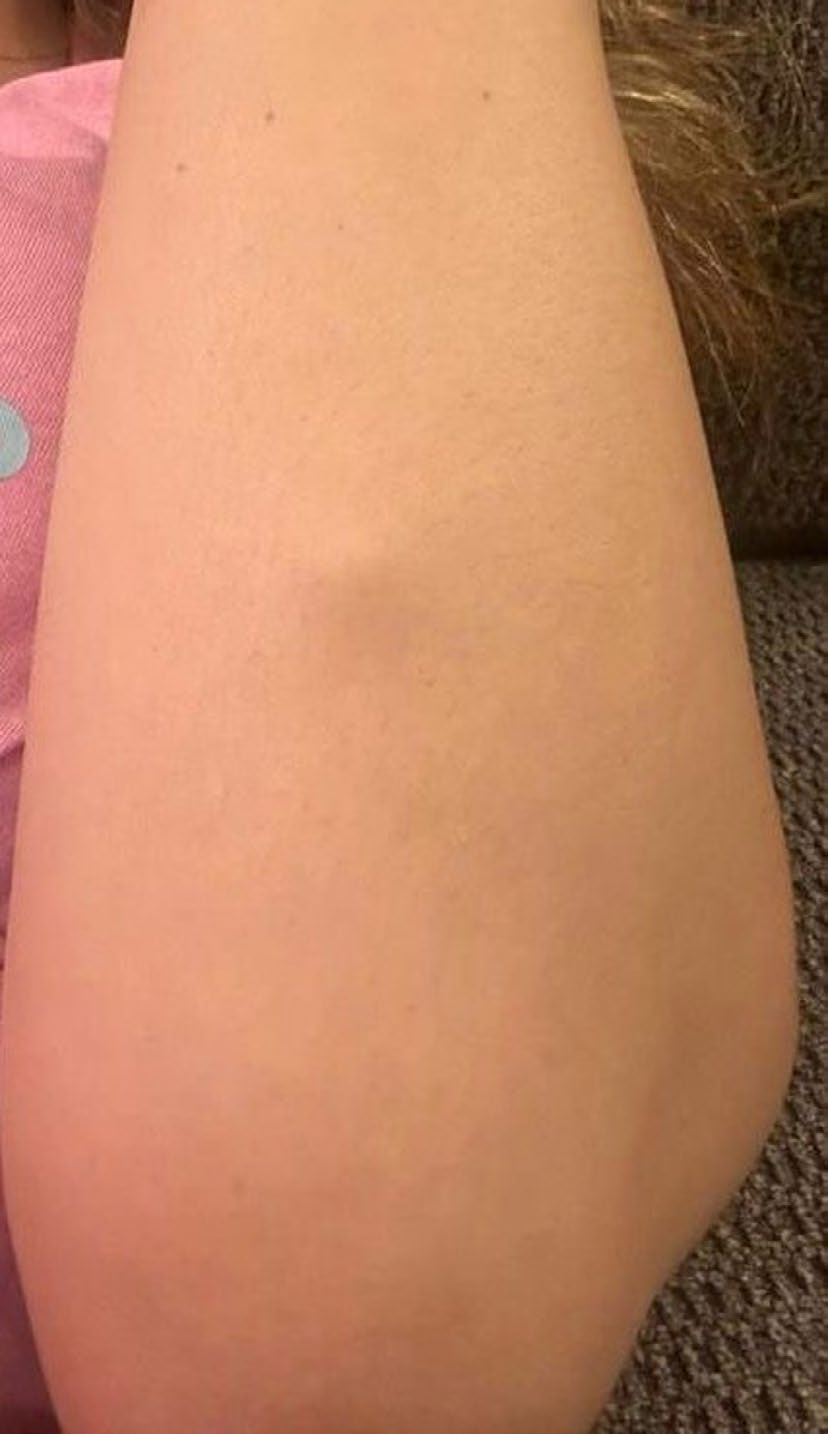
Clinical aspect of the soft tissue mass.

A 33‐year‐old female patient presented to the clinic with a painful soft tissue mass on the dorsal aspect of the left mid‐forearm. The signs were noticed 8 months prior to her consultation. Physical examination revealed a 2 × 2 cm firm and tender mass slightly mobile under the skin (Figure 1). The skin color was normal with no signs of inflammation around the mass. The patient reported a rapid increase in size causing discomfort and pain during the last 2 months. The pain was exaggerated on movements, mainly with extending the wrist.

### 2.2 Differential Diagnosis

The clinical aspect and location pointed at first to a benign soft tissue tumor. The differential diagnosis was either a lipoma, atypical lipomatous tumor, or a cystic lesion. However, due to the rapid size evolution, a decision was made to excise the tumor.

## 3 Methods

**FIGURE 2:**
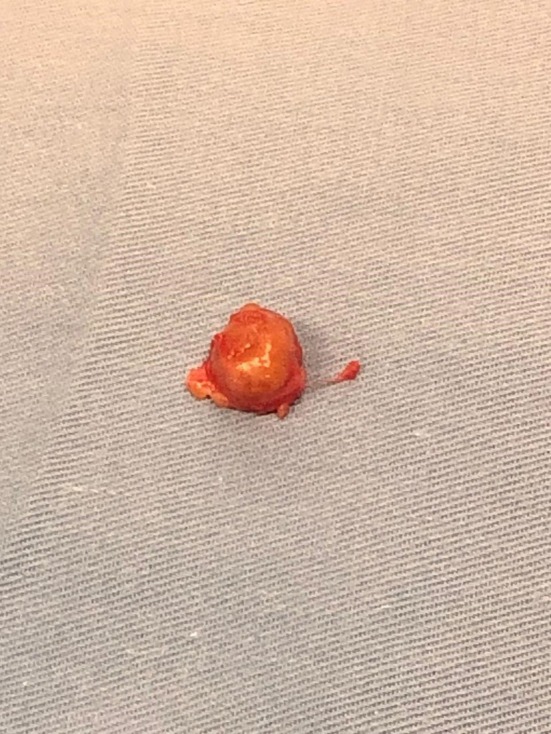
Postoperative image of the excised tumor.

An incision was made over the identified mass, and the tumor was found deep beneath the antebrachial fascia, intramuscular within the fibers of the extensor muscles. Dissection was carried out around the tumor, and an “en bloc” resection was performed, and the specimen was sent to pathology (Figure 2). The mass was not associated with any adjacent tendons. The antebrachial fascia was sutured, and subcutaneous closure of the wound was completed.

## 4 Conclusion and Results

**FIGURE 3:**
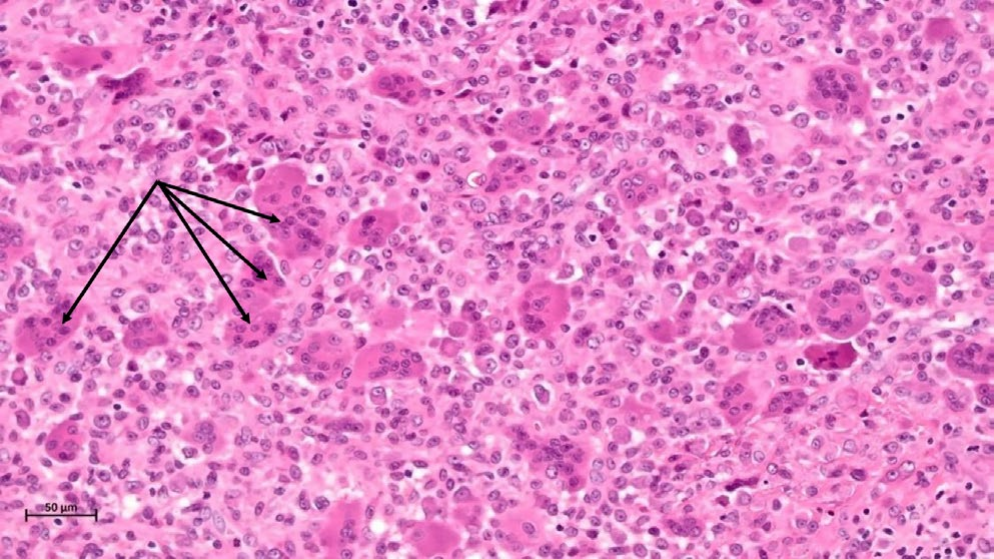
Microscopic aspect under 5× magnification showing the well‐demarcated lesion with a fibrous capsule (arrows: Multinucleated cells).

**FIGURE 4:**
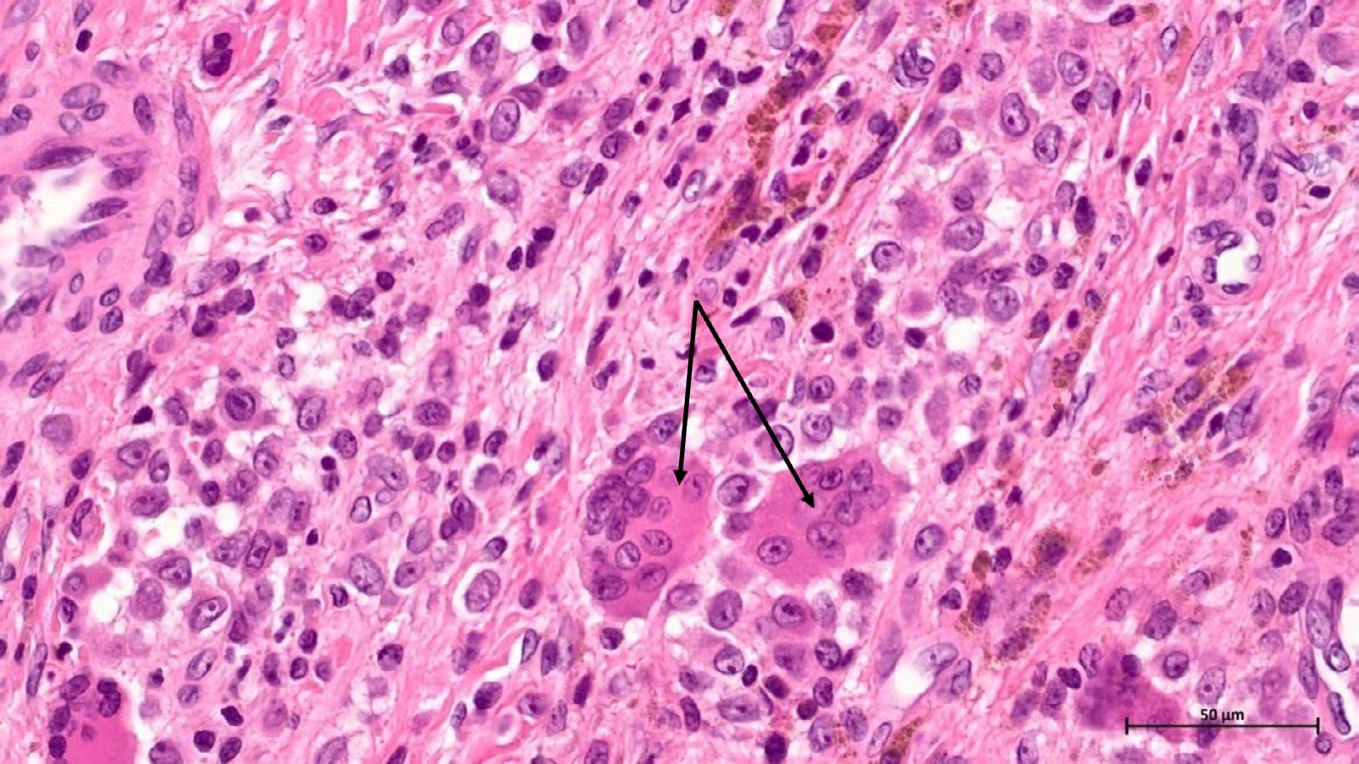
Microscopic aspect under 20× magnification showing the multinucleated giant cells and the mononuclear cells (arrows: Multinucleated cells).

**FIGURE 5:**
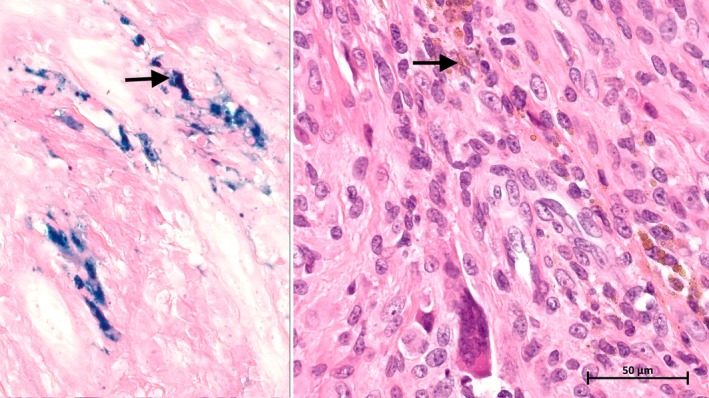
Microscopic image of Hemosiderin deposition on H&E stain and Perl's Blue Iron stain (40× magnification).

The histological exam revealed a giant cell tumor of soft tissue with no capsular invasion and margins that were negative. The lesion was well circumscribed with no infiltrative borders. It was composed of mononuclear cells and multinucleated giant cells (Figures 3 and 4). Hemosiderin deposits were noted (Figure 5).

**FIGURE 6:**
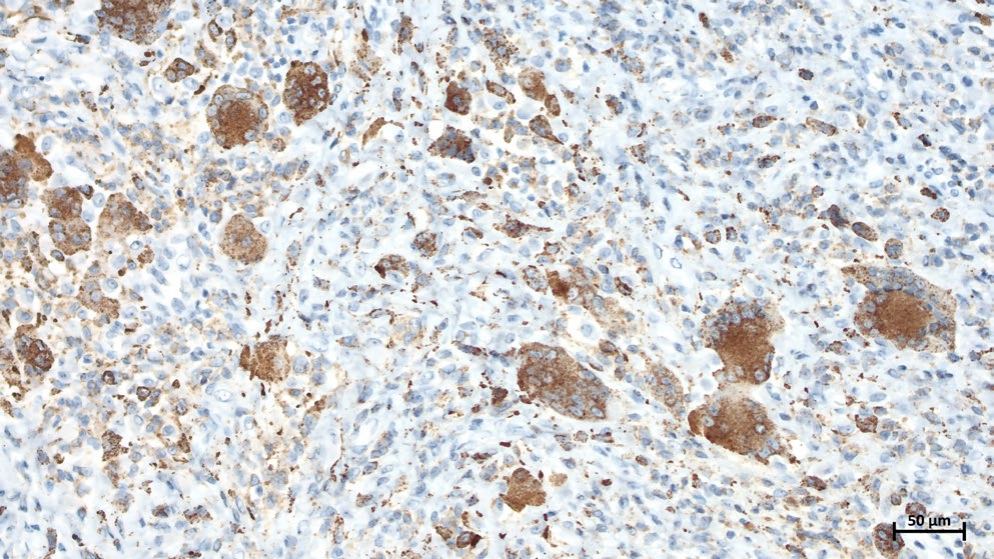
Microscopic image of CD68 immunohistochemical staining in multinucleated giant cells and some of the mononuclear cells (20× magnification).

**FIGURE 7:**
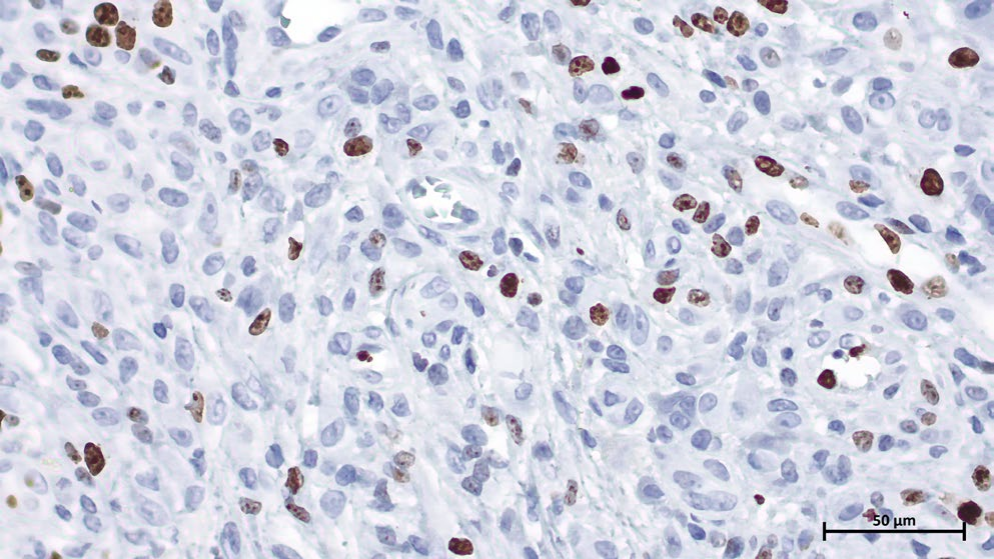
Microscopic image of Ki‐67 (MIB‐1) immunohistochemical staining showing the proliferative activity in the mononuclear cells (40× magnification).

Immunohistochemical staining showed the multinucleated giant cells and some of the mononuclear cells to be positive for CD68. PanCytokeratin (AE1/AE3), S100, and P63 were negative in all the lesion components. The mononuclear cells showed a proliferative index (Ki‐67) up to 20% (Figures 6 and 7). The patient was informed about the potential risk of recurrence, and regular follow‐up appointments were recommended. At the last follow‐up, 6 months after resection, there was no clinical evidence of recurrence. Informed consent was obtained from the patient before submission allowing the authors to publish clinical, imagery, and histologic photos.

This case contributes to a better understanding of GCT‐ST, particularly the presentation variability in terms of anatomical locations with deep‐seated positions. The variability in clinical presentation, combined with the benign histological features, underscores the importance of careful diagnostic and surgical management to ensure favorable outcomes. This case contributes to the limited literature on GCT‐ST in atypical locations, further emphasizing this tumor's variability in clinical presentation.

## 5 Discussion

GCT‐STs are uncommon tumors that most often occur in the extremities, with the lower limbs being more frequently affected than the upper limbs. In Oliveira et al.'s study of 22 primary GCT‐ST cases, the upper limbs were the least frequently involved (3/22 cases), with the lower limbs being the most common location (11/22 cases), followed by the trunk (7/22 cases) [4]. The review published by Hasan et al. showed that the face could be another location for GCT‐ST [5].

Most superficial tumors were described as superficial and confined to the dermis [4]. With 86.4% of their cases being superficial, these authors reported only 1 case (4.5%) of GCT‐ST involving the forearm, that was described as superficial subcutaneous tumor [4]. We are aware of another case of GCT‐ST located superficially in the forearm; these authors described a superficial hyperpigmented papule that grew rapidly on the forearm skin with gross signs of ulceration [5].

Therefore, the atypical forearm location of our case added to the deep‐seated intramuscular presentation could be considered exceptional.

The main symptomatology of our case was classical for GCT‐ST, as most patients consult for a growing mass sign. On the other hand, the mass was painful, which has been shown to be a much less common symptom; only 2 patients out of 22 (9%) experienced pain, as reported by Oliviera et al. [4].

Macroscopically, our case showed the typical aspect of a GCT‐ST characterized by a well‐circumscribed multi‐lobulated solid mass [6]. Microscopically, as in most benign GCT‐STs, the tumor showed a multinodular architecture where cellular nodules separated by fibrous septa were bathing in a richly vascularized stroma. Unlike other rare GCT‐STs [2, 4, 7, 8], features such as stromal hemorrhage or vascular invasion were not observed. It is noteworthy that signs of vascular invasion that are reported in a few cases are not indicative of poor prognosis or metastatic potential in benign GCT‐STs [4, 9].

In our case and similarly to the reported cases of small GCT‐ST, imaging is often not conducted before excision. Since the initial diagnosis was a lipoma, no biopsy has been performed. Nevertheless, we followed the guidelines and removed the tumor as “en bloc” resection aiming for final diagnosis and treatment. Although imaging such as radiographs, ultrasound, and CT Scan could provide valuable information, insights into the tumor's behavior and its relationship with surrounding structures are best provided by MRI [10, 11]. Commonly, MRI findings show low to intermediate signal intensity on T1‐weighted images and variable intensity on T2‐weighted images. On the other hand, positron emission tomography (PET) imaging of GCT‐ST shows increased fluorodeoxyglucose (FDG) uptake, which can lead to false‐positive diagnoses of malignancy [2, 12].

In our case, since the initial diagnosis was a lipoma, no imaging was conducted.

Complete surgical excision is the preferred treatment for GCT‐ST, as local recurrence rates can be as high as 21%, particularly in cases of incomplete resection [2]. However, Ichikawa et al. recorded that the recurrence rate for deep GCT‐ST could approach 50% even after surgical excision with negative margins [13]. For this reason, some authors recommend combining surgery with adjuvant radiation therapy; however, the indications for this approach are not yet well established [2, 5, 14, 15]. There is a need for comparative studies to better define the role of radiation therapy in the treatment of this tumor.

## Author Contributions

**Kaissar Yammine:** conceptualization, data curation, investigation, methodology, supervision, validation, writing – original draft, writing – review and editing. **Emanuel‐Youssef Dib:** resources, writing – original draft, writing – review and editing. **Moueen Bou Ghanem:** methodology, visualization, writing – original draft, writing – review and editing.

## Ethics Statement

The authors have nothing to report.

## Consent

Written informed consent was obtained from the patient to publish this report in accordance with the journal's patient consent policy.

## Conflicts of Interest

The authors declare no conflicts of interest.

## Data Availability

Data available on request from the authors.
